# Plant Architecture, Tolerances to NaCl and Heavy Metals May Predispose *Tragus racemosus* to Growth Around Motorways

**DOI:** 10.3390/plants14050784

**Published:** 2025-03-04

**Authors:** Božena Šerá, Marianna Molnárová, Mustafa Ghulam, Pratik Doshi, Hubert Žarnovičan

**Affiliations:** Department of Environmental Ecology and Landscape Management, Faculty of Natural Sciences, Comenius University Bratislava, Mlynská dolina, Ilkovičova 6, 842 15 Bratislava, Slovakia; marianna.molnarova@uniba.sk (M.M.); ghulam1@uniba.sk (M.G.); pratik.doshi@uniba.sk (P.D.); hubert.zarnovican@uniba.sk (H.Ž.)

**Keywords:** heavy metals, contaminated soil, NaCl, road, seed, reproduction

## Abstract

*Tragus racemosus* often grows in close proximity to motorways. The aim of this work was to determine whether the seeds of the species can grow under salt (NaCl) stress, how the plants are able to accumulate heavy metals and what plant architecture prerequisites they have for spreading. It was found that the structure of the plant consists of a single rosette of the first order, from which shoots of the first order develop, on which rosettes of the second order grow, and this is repeated modularly. Higher-order rosettes can produce their own root systems. Research on this species revealed its small salt and heavy metal tolerances during germination and early development. The concentration of metals in the above-ground parts of plants was of the following rank: Fe >> Zn > Ni ≥ Pb > Cu; for soil, it was Fe >> Pb > Cu > Ni. The plant germinates successfully and grows in environments containing NaCl up to 0.50% (including solutions of 0.12% and 0.25%). However, higher salt contents of 0.99% and 1.96% proved lethal for seed germination. This tolerance to salt explains why *T. racemosus* commonly grows along motorways where winter road maintenance involves the application of salt. These adaptations give the species a competitive advantage in these human-modified environments. Furthermore, *T. racemosus* turned out to be a possible Ni hyperaccumulator.

## 1. Introduction

*Tragus racemosus* (L.) All. is a relatively rare member of the Poaceae family, recently observed primarily along roads and motorways [1,2,3,4] in Central Europe. This species is native to southern, western and southwestern Europe [5,6] and southwestern Asia and Africa [7]. Confirmed occurrences outside Europe have been documented in both North and South America [7,8].

During faculty research conducted on permanent plots at a grade-separated junction near Selenec village (Nitra district, Slovakia), a population of *T. racemosus* was discovered in 2021 growing in distance close to the asphalt motorways [9]. Since then, its population has been steadily increasing [10].

Because soil near motorways is often found with high NaCl content due to salting during the winter season and is contaminated with heavy metals, not all plant species can tolerate these stresses. As Kumar et al. [11] stated, there is no clear evidence to suggest that plants have a specialized root exudation process explicitly aimed at defending against salt stress. However, numerous studies have shown that when a plant is subjected to salt stress, it releases root exudates [12]. It is known that crop plants belonging to the Poaceae family can release root exudates, such as phytosiderophores, in the case of metal deficiency for their uptake into roots, which Prasad et al. [12] described in their study.

Grasses are known to be tolerant to higher concentrations of heavy metals [13], and plant species of the Poaceae family especially have the potential for heavy metal bioaccumulation [14]. Plant species with a bioconcentration factor (BCF) and a tolerance index Ti > 1 are considered suitable for phytoextraction. Members of the grass family and, therefore, of the Poaceae family are recommended for the restoration of contaminated lands, as mentioned in Patra et al. [14].

The objectives of this study are (a) to describe some plant architecture features that predispose this species to spread along roads and highways, (b) to determine reproductive capacity, (c) to expand our knowledge on salt (NaCl) stress management in germinating *T. racemosus* seeds and (d) to analyze plants’ ability to accumulate heavy metals under real soil conditions. The results of this study provide original data that help us understand the strategy of the species and help explain why *T. racemosus* can successfully grow in the specific conditions around roads and highways.

## 2. Results

### 2.1. Plant Morphology and Reproductive Capacity

**Figure 1:**
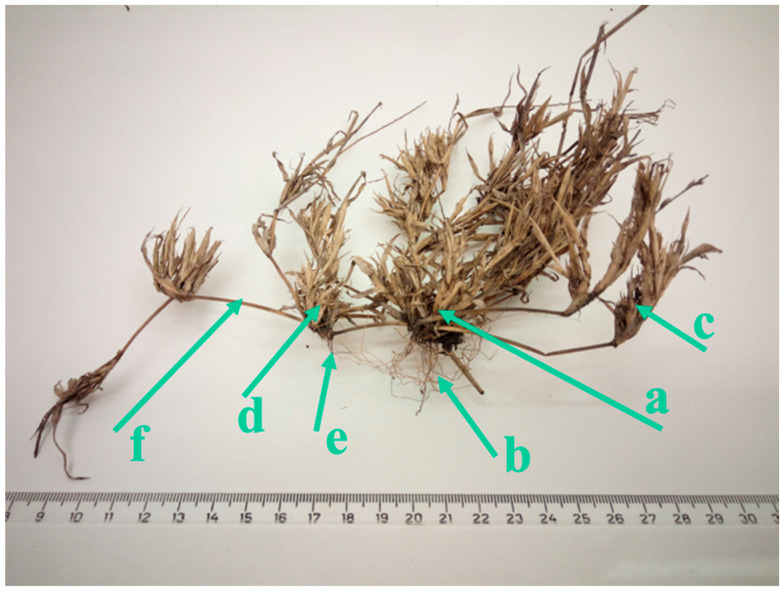
*T. racemosus*—plant architecture (a—rosette of the 1st order, b—root system of the rosette of the 1st order, c—shoot of the 1st order, d—rosette of the 2nd order, e—root system of the rosette of the 2nd order, f—shoot of the 2nd order).

The plant exhibits a distinct hierarchical structure, comprising a primary (first order) rosette from which primary shoots emerge. These shoots bear secondary (second order) rosettes, which in turn produce secondary shoots. The species shows the ability to spread capability, as the primary rosette produces first-order shoots with secondary rosettes that can develop their own root systems (Figure 1). Research findings revealed that a single plant typically has a diameter of 31.3 ± 5.6 cm, primary shoots of 13.0 ± 2.5 cm, secondary shoots of 24.4 ± 12.7 cm and fertile shoots of 6.5 ± 1.7 cm. Fertile shoots bearing spikes can develop from all rosettes. Each spike contains 42.0 ± 18.2 grains, and each fertile shoot terminates in 3 ± 1 spikes. The reproductive capacity of *T. racemosus* was calculated at 819 ± 42 grains per plant.

### 2.2. Heavy Metals in Plant Biomass and Soil

For As, Cd, Cr, Hg, Mn, Sb and Se, we did not confirm the presence of these elements in soil or plants, including others introduced in Table 1. The concentration of metals in the plants are ordered in the following rank: Fe >> Zn > Ni ≥ Pb > Cu and for soil as Fe >> Pb > Cu > Ni. The relevant bioaccumulation detected was only for nickel, while no significant accumulation of Fe, Pb and Cu was calculated. The highest concentration in the soil was determined for iron (52.783 g kg^−1^ DM), but bioaccumulation in *T. racemosus* was very low, and the bioaccumulation factor was close to zero (0.07). Although copper and lead accumulated in the plant in lower concentrations (BCF < 1, Table 1), nickel was 3.64 times higher in the plant compared to the soil. The BCF value was greater than 1, which could be a potential plant for the accumulation of this heavy toxic metal. Moreover, we observed the presence of Zn in the plant but not in the soil.

### 2.3. Salt Stress Test via Seed Germination

**Figure 2:**
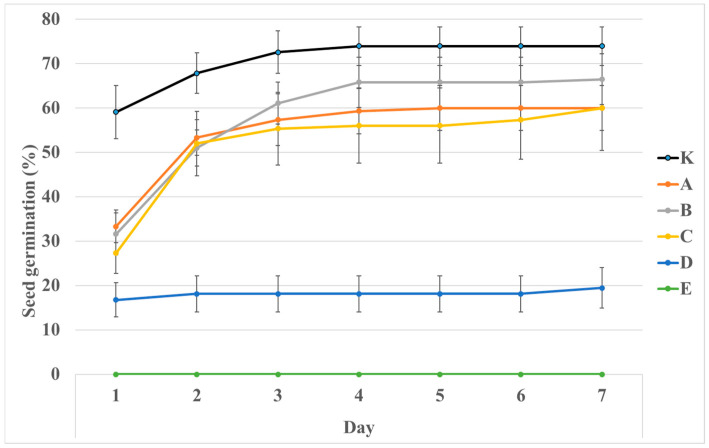
Seed germination (%) of *T. racemosus* under stress with different content of NaCl (K—0%, A—0.12%, B—0.25%, C—0.50%, D—0.99%, E—1.96%).

All seeds started to germinate at the same time except the seeds treated with the 1.96% NaCl solution, which did not germinate at all (Figure 2). The seed set that was treated with 0.99% NaCl showed very low germination, which was 20% at the end of the experiment. Seed sets treated with NaCl solutions of 0.12%, 0.25% and 0.50% germinated worse compared to the control, but no significant differences were observed. The highest germination was recorded in seeds that were not treated with NaCl solution (content 0%, control set), where it did not change from the fourth day and was at a value of 74%. The course of seed germination is recorded in Figure 2 and Table 2.

For most of the characteristics monitored, a significant difference was found between the groups of sets K, A–C (treated with NaCl solution 0.00% to 0.50%) and treatments D and E (solutions 0.99% to 1.96%) (Table 2, Table 3 and Table 4). Furthermore, for the germination index, a significant difference was found between the control and treatments A–C; on the contrary, for the root-to-shoot length ratio, a significant difference was also found between K and C. The highest values were usually recorded for the characteristic of the control set; for the root-to-shoot length ratio, it was the lowest value. An overview of the monitored characteristics is provided in Table 3 and Table 4.

## 3. Discussion

### 3.1. Plant Architecture and Reproductive Capacity

The population of *T. racemosus* has been monitored in the habitat since 2021 (cca. 20 individuals). In 2022, the population increased to approximately 130 individuals [9]. By 2023, the population will occupy an area of approximately 1.5 m × 25 m, with 70% coverage and approximately 200 individuals [10]. The successful population growth is probably attributed to the high reproductive capacity of the plant (819 ± 42 seeds/plant) and its ability to spread vegetatively, including the rooting of secondary rosettes (Figure 1).

During autumn, when the annual *T. racemosus* completes its life cycle, a “tumbleweed effect” [15] was observed in our habitat. Dry above-ground parts of several plants were seen moving along the road and in close proximity to the asphalt, propelled by air currents generated by passing vehicles. The movement of vehicles probably helps to transport these above-ground parts (containing mature achenes) in the direction of traffic flow, away from the original population. Therefore, road traffic may facilitate the spread of this species along roads and motorways.

### 3.2. Heavy Metal Analyses

Although to our knowledge, *T. racemosus* has not already been described in the literature as heavy metal hyperaccumulators, Poaceae plant species are known metal hyperaccumulators of Al, As, Cd, Cr, Cu, Ni, Pb and Zn ions and can be potential phytoremediators [14].

The concentration of Fe can be relatively high in many localities, such as the waste dump site of the iron mine at Joda East Iron mine in Odisha, India [16], where the concentration of Fe in the soil is 1725.28 ± 48.32 mg kg^−1^. However, near an iron and copper mine in the central part of Iran in the Hamadan province, the Fe concentration was measured as 32,466 ± 1565 mg kg^−1^ of soil [17], which was comparable to our value in Table 1. Because the studied motorways are relatively new and the material used for their construction can contain relatively high concentrations of metals, the contamination of the soil near roads can be suitable only for plants that can tolerate higher metal concentrations. Furthermore, the use of salt for the winter maintenance of roads has also increased the salt concentration. Nouri et al. [17] measured the following concentrations (mg of metal/kg of DM above ground) in *Stipa barbata* Desf. as a species of the Poaceae plant: 329.3 for Zn; approximately 6000 for Fe; 100 for Mn; 72 for Cu; and transportation factor (TF) as TF (Fe) = 1.23, TF (Mn) = 0.19, TF (Zn) = 1.04 and TF (Cu) = 0.78.

The bioconcentration of Fe in the above-ground parts was minimal because the BCF was equal to 0.07. *T. racemosus* is likely able to moderate the entry of this metal into the roots and avoid its phytotoxic effect, but Fe can be helpful to photosynthesis. Wong et al. [18], in their study, observed that wheat in the presence of higher concentrations of NaCl and Fe had higher concentrations of chlorophyll a and chlorophyll band and an increase in the dry mass of the roots and shoots together with the leaf area. The crucial role in iron homeostasis under abiotic stress can also play the role of ferric reductase oxidase (FRO), which was observed in *Gossypium* sp. [19]. This enzyme is essential for iron uptake and homeostasis in plants. For example, plants belonging to the Poaceae family, such as barley or wheat, can release phytosiderophores from their roots, which help in the uptake of Zn under conditions of Zn deficiency, thus supporting the growth of monocotyledonous crops [12]. Although no Zn was detected in the soil (Table 1), it could partially explain the high concentration in *T. racemosus*. It is possible that Zn concentrations below the detection limit of our electrochemical method were present in the studied locality. It is known that siderophores, together with metallophores, have been extensively studied for their possible role in fighting environmental pollution [20].

### 3.3. Salt Stress Test

The germination test under NaCl stress conditions confirmed that too high salinity (1.96% NaCl) killed the seeds (Figure 2, Table 2). The highest values of the monitored characteristics were generally recorded in the control set, while the lowest value was recorded for the root-to-shoot length ratio characteristic (Table 4). This indicates that NaCl reduced the ability of the seeds to germinate and grow in contaminated substrate conditions.

Nováková et al. [21] investigated the effect of the distance of the plant from the asphalt on its growth and reproduction. This study found that although the soil was not classified as saline, proximity to the road had a negative impact on the plants. Specifically, the root and shoot biomass of both species was significantly lower in roadside specimens compared to control plants. The differences were more pronounced in roots, with both length and dry matter being significantly lower in roadside specimens. In *Echinochloa crus-galli* (L.) P. Beauv., these differences were evident up to 0–50 cm from the road, while in *Digitaria sanguinalis* (L.) Scop. The impact extended up to 100 cm. Beyond these distances, the differences in shoot length were not statistically significant.

The distance between the plant and the asphalt is an important factor. The soil near asphalt is more affected by traffic and maintenance, affecting both soil salinity and the content of some heavy metals. Therefore, further research could focus on perpendicular gradients to the road and the effect of distance from the road on both plant growth and soil parameters.

## 4. Materials and Methods

### 4.1. Habitat Characteristics

Plants and fruits (caryopses, grains) of the *T. racemosus* plant were harvested in the autumn of 2023 at an interchange near the village of Selenec (Slovakia, east of Nitra town). The conductivity of the surface soil layer (5–10 cm) at the location of *T. racemosus* occurrence was 3.3 ± 0.5 mS cm^−1^. A brief description of the site is given in Table 5.

### 4.2. Plant Architecture and Reproductive Capacity

Ten randomly selected specimens were harvested, and for each specimen, the rosette was measured, plant branching was documented and the number of spikes was counted. For three randomly selected spikes, the number of fruits was determined. From these data, the following average values (mean ± SD) were calculated: rosette diameter, number of main branches, number of secondary branches, total number of branches and the number of spikes and fruits per plant. The reproductive capacity of a plant was determined as the number of grains produced in one year by one individual plant.

### 4.3. Heavy Metal Analyses (in Plant and in Soil)

Five random soil samples (50 g each, 0–5 cm depth) and five randomly occurring plants (above-ground part) were collected at the study site using standard methods. Plants were washed with distilled water before further processing. The soil and plant samples were dried at room temperature in laboratory. For determination of (semi)metals (arsenic—As, cadmium—Cd, chromium—Cr, copper—Cu, iron—Fe, mercury—Hg, manganese—Mn, nickel—Ni, lead—Pb, antimony—Sb, selenium—Se, zinc—Zn), the above-ground part of the plant was used. The weights of 52.8 mg of plant dry mass (DM) and 325.3 mg of dried soil were used for mineralization in 5 mL of HNO_3_:H_2_O_2_ (4:1) mixture during night in ZA–1 autoclave (Czech Republic) [25]. On the next day, the samples were mineralized in a thermostat at 180 °C for 1 h. Then, the mineralized samples were cooled at laboratory temperature, filled up to 25 mL with distilled water and (semi)metals were measured by galvanostatic chronopotentiometric electrochemical method on the EcaFlow 150GLP [19]. Metal ions (M) are electrochemically deposited from the flowing sample solution in the porous working electrode [26]. Deposition is carried out by applying a suitable deposition current. In the next step, the deposit is stripped galvanostatically, whereas the stripping chronopotentiogram is recorded and evaluated. The method measures metals mostly in the concentration range of 0.5–1000 µg L^−1^, and the reproducibility for Pb is 1.5% at 50 µg L^−1^ in the measured solution. The method is comparable to atomic absorption spectrometry (AAS) in the sensitivity, accuracy, precision and reliability of the measured results. The bioconcentration factor (BCF) for (semi)metals was then calculated by the Formula (1) using average values for metal concentrations:

(1) $$BCF=\frac{metal\;concentration\;in\;the\;above-groud\;part\;of\;plant}{metal\;concentration\;in\;the\;soil}$$

### 4.4. Salt Stress Test via Seed Germination

The fruits of *T. racemosus* for the NaCl salt stress germination test were collected from randomly selected samples, totaling a minimum of 2000 fruits. The following NaCl concentration solutions were prepared using distilled H_2_O: K (control)—0%, A—0.12, B—0.25, C—0.50%, D—0.99, E—1.96. The experiment was carried out using plastic Petri dishes (9 cm in diameter), each containing three filter papers, 6 mL of solution and 30 seeds. Each variant was replicated five times; thus, each treatment (K, A–E) utilized five Petri dishes and 150 fruits. The seeds germinated in a cultivation chamber (darkness, temperature 25 °C) for seven days. The number of germinated seeds was measured daily. Seed germination (%), germination index, root length (mm), shoot length (mm), seedling fresh weight (mg), seedling dry matter weight (mg), seedling vigor index I (mm), seedling vigor index II (mg), seedling vigor index III (mg) and root-to-shoot length ratio were determined for each variant in the last day of cultivation according to Šerá [27].

### 4.5. Data Analyses

The germination data were analyzed using statistical package STATISTICA 14.0.0.15. [28]. One-way analysis of variance (ANOVA) was performed, followed by Tukey’s HSD test for multiple comparisons among treatments (categorical variable was treatment with these options K, A–E). Statistical calculations were conducted at α < 0.5.

## 5. Conclusions

The ecological strategy of *Tragus racemosus*, a species found along motorways, is characterized by three main adaptations: plant architecture (a system of shoots that can root), high grain production, tolerance to contaminated soils (including salt NaCl and heavy metals such as Fe, Cu and Pb) and effective dispersal mechanisms from the parent plant through vegetative growth and spreading as a steppe runner. It seems that *T. racemosus*, similar to other species in the Poaceae family, is able to accumulate high amounts of these heavy metals in its shoots, and this strategy likely helps growth in the soil with higher amounts of not only salt but also metals. Although the presence of Fe, Pb and Cu was relatively low in the plant, the bioconcentration factor for Ni was 3.64, referring to *T. racemosus* as a possible nickel hyperaccumulator. Due to our measurements, we consider this plant to be suitable for growing in soils with high metal concentrations (mainly Fe, Cu and Pb) and as a suitable candidate for nickel hyperaccumulation, which can be used in phytoremediation processes. The species shows potential as a nickel hyperaccumulator and could be valuable for phytoremediation applications.

## Figures and Tables

**Table 1:** Concentrations of (semi) metals in soil, *T. racemosus* and their bioconcentration factor (BCF). Average values with their standard errors of mean (SE are inserted, n = 3. Legend: DM—dry mass; nd—not detectable; * ions are determined together during same measurement (Istran Company introduced only information of Pb producibility).

(Semi)Metal	As	Cd	Cr	Cu	Fe	Hg
Soil (mg kg^−1^ DM)	nd	nd	nd	6.59 ± 0.43	52,783 ± 1416	nd
*T. racemosus* (mg kg^−1^ DM)	nd	nd	nd	1.57 ± 0.09	3715 ± 323	nd
BCF	-	-	-	0.24	0.07	-
Concentration range (µg L^−1^)	0.5–200	0.5–1000	2–200	0.5–1000	10–500	0.1–1000
Reproducibility	4.2% at 50 µg L^−1^	1.5% at 50 µg Pb L^−1^ *	1.8% at 100 µg L^−1^	1.5% at 50 µg Pb L^−1^ *	1.5% at 200 µg L^−1^	2.0% at 50 µg L^−1^
**(Semi)Metal**	**Mn**	**Ni**	**Pb**	**Sb**	**Se**	**Zn**
Soil (mg kg^−1^ DM)	nd	4.37 ± 0.05	20.53 ± 3.26	nd	nd	nd
*T. racemosus* (mg kg^−1^ DM)	nd	15.92 ± 0.36	14.30 ± 1.44	nd	nd	19.74 ± 1.66
BCF	-	3.64	0.7	-	-	-
Concentration range (µg L^−1^)	1–500	1–200	0.5–1000	0.5–500	0.5–500	0.5–1000
Reproducibility	1.5% at 50 µg L^−1^	2.5% at 50 µg L^−1^	1.5% at 50 µg Pb L^−1^ *	3.2% at 50 µg L^−1^	3.2% at 50 µg L^−1^	1.5% at 50 µg Pb L^−1^ *

**Table 2:** Measured characteristics of seed germination in *T. racemosus* after salinity stress in the last day of seed cultivation. SE: standard error; HSD: results of Tukey’s HSD test; significant differences at *p* < 0.05 are indicated by different letters.

	NaCl (%)	Seed Germination (%)			Germination Index		
		Mean	SE	HSD	Mean	SE	HSD
K	0.00	73.931	4.820	a	51.610	3.944	a
A	0.12	60.000	5.676	a	37.355	3.226	d
B	0.25	66.460	6.475	a	37.982	4.081	d
C	0.50	60.000	3.801	a	34.531	2.285	d
D	0.99	19.471	1.273	b	13.659	0.646	b
E	1.96	-	-	-	-	-	-

**Table 3:** Measured characteristics of early growth in (weights and lengths) *T. racemosus* after salinity stress in the last day of seed cultivation. SE: standard error; HSD: results of Tukey’s HSD test; significant differences at *p* < 0.05 are indicated by different letters.

	NaCl (%)	Seedling Fresh Weight (mg)			Seedling Dry Matter Weight (mg)			Shoot Length (mm)			Root Length (mm)		
		Mean	SE	HSD	Mean	SE	HSD	Mean	SE	HSD	Mean	SE	HSD
K	0.00	1.637	0.116	a	0.214	0.018	a	11.173	0.827	a	36.073	2.657	a
A	0.12	1.395	0.109	a	0.156	0.011	a	9.127	0.796	a	30.733	2.423	a
B	0.25	1.556	0.117	a	0.189	0.008	a	9.577	0.941	a	33.749	3.430	a
C	0.50	1.332	0.078	a	0.182	0.010	a	7.287	0.407	a	27.713	1.539	a
D	0.99	-	-	-	-	-	-	-	-	-	-	-	-
E	1.96	-	-	-	-	-	-	-	-	-	-	-	-

**Table 4:** Measured characteristics of early growth (seedling vigour indexes, root-to-shoot length ratio) in *T. racemosus* after salinity stress in the last day of seed cultivation. SE: standard error; HSD: results of Tukey’s HSD test; significant differences at *p* < 0.05 are indicated by different letters.

	NaCl (%)	Seedling Vigor Index I (mm)			Seedling Vigor Index II (mg)			Seedling Vigor Index III (mg)			Root-to-Shoot Length Ratio		
		Mean	SE	HSD	Mean	SE	HSD	Mean	SE	HSD	Mean	SE	HSD
K	0.00	3557.856	490.040	a	123.246	16.877	a	15.942	1.983	a	3.230	0.066	a
A	0.12	2464.033	420.070	a	86.184	14.533	a	9.582	1.594	a	3.378	0.079	ab
B	0.25	2990.579	531.397	a	106.324	16.535	a	12.695	1.677	a	3.523	0.059	ac
C	0.50	2125.356	238.145	a	80.891	9.131	a	11.016	1.128	a	3.804	0.006	d
D	0.99	-	-	-	-	-	-	-	-	-	-	-	-
E	1.96	-	-	-	-	-	-	-	-	-	-	-	-

**Table 5:** Basic characteristics of the habitat of *T. racemosus*.

Locality	Nitra Selenec, Feeder to Expressway R1, Direction Towards Trnava, in Operation Since 2011
Coordinates	48°18′36.33″ N, 18°08′25.49″ E
Description of habitat	Left edge of the road under the guard rail
Altitude (m a.s.l.)	161
Exposition	SE-S
Slope (°)	10
Geomorphological unit [22]	Podunajská pahorkatina
Climatic region [23]	Warm region, 50 or more summer days annually on average (with daily maximum air temperature ≥ 25 °C)
Mean annual precipitation total (mm) [24]	500–600

## Data Availability

All data generated and analyzed during this study are available from the corresponding author upon reasonable request.
